# Supplementary material for: Extracellular Glycolytic Activities in Root Endophytic Serendipitaceae and Their Regulation by Plant Sugars
Source: Microorganisms. 2022 Jan 29;10(2):320. doi: 10.3390/microorganisms10020320 (PMC8878002; doi:10.3390/microorganisms10020320)
Supplement: Supplementary file 1 [file microorganisms-10-00320-s001.zip › microorganisms-1545195-supplementary.pdf]

Table S1. Activities of glycolytic enzymes (nkat/gr) in the cytosol of *S. indica* when cultivated with glucose (Glu), fructose (Fru), sucrose (Suc), an equimolar mix of glucose and fructose (Glu+Fru), mannose (Man) and without sugar for 14 days. The table shows average values ± standard errors. Significant differences of enzymatic activities in mycelia fed with the different sugars are indicated by different letters (Tukey HSD test; $n = 6$). Significant higher activities compared to starvation (no sugar) are indicated in green, lower activities in red bold letters.

S. indica	**Glu**	**Fru**	**Suc**	**Glu+Fru**	**Man**	**No sugar**
	Enzymatic activities [nkat/g FW]					
**Aldolase**	0.37^{b} ± 0.04	**1.26^{a}** ± 0.04	0.46^{b} ± 0.01	0.37^{b} ± 0.00	**1.16^{a}** ± 0.03	0.47^{b} ± 0.05
**Fructokinase**	5.07^{c} ± 0.63	11.84^{b} ± 0.87	7.21^{c} ± 0.46	4.93^{c} ± 0.13	**16.73^{a}** ± 0.98	1.40^{d} ± 0.11
**G6PDH**	0.06 ± 0.03	0.01 ± 0.01	0.02 ± 0.02	0.02 ± 0.01	0.05 ± 0.03	0.00 ± 0.00
**GP**	0.06^{c} ± 0.01	**0.26^{a}** ± 0.01	**0.01^{d}** ± 0.01	0.08^{c} ± 0.01	**0.27^{a}** ± 0.01	0.15^{b} ± 0.01
**Hexokinase**	5.70^{c} ± 0.23	10.20^{b} ± 0.50	6.24^{c} ± 0.64	4.99^{c} ± 0.44	**18.53^{a}** ± 1.20	6.35^{c} ± 0.52
**Invertase pH 4.5**	34.16^{bc} ± 0.20	54.04^{b} 8.85	39.04^{bc} ± 5.23	**22.46^{c}** ± 0.29	**131.21^{a}** ± 5.90	50.43^{b} ± 4.36
**PFK**	0.01^{c} ± 0.00	0.13^{b} 0.01	0.054^{b} ± 0.01	0.04^{c} ± 0.00	**0.63^{a}** ± 0.04	0.03^{c} ± 0.01
**PGI**	1.63^{b} ± 0.06	1.09^{b} ± 0.13	2.36^{b} ± 0.18	1.89^{b} ± 0.09	0.93^{b} ± 0.05	71.05^{a} ± 1.27
**PGM**	11.29^{a} ± 0.02	7.82^{b} ± 0.19	11.13^{a} ± 0.12	8.69^{b} ± 0.21	**5.14^{c}** ± 0.30	10.79^{a} ± 0.84
**UGPase**	3.93^{c} ± 0.02	**11.60^{a}** ± 0.65	6.40^{b} ± 0.64	6.42^{b} ± 0.26	**12.50^{a}** ± 0.10	1.78^{d} ± 0.39

G6PDH: glucose-6-phosphate dehydrogenase; GP glycogen pyrophosphorylase; PFK: phosphofructokinase; PGI: phosphoglucose isomerase; PGM: phosphoglucose mutase; UGPase: UDP-glucose pyrophosphorylase.

Table S2. Activities of glycolytic enzymes (nkat/gr) in the cytosol of *S. herbamans* when cultivated with different sugars: glucose (Glu), fructose (Fru), sucrose (Suc), an equimolar mix of glucose and fructose (Glu+Fru), mannose (Man) and no sugar for 14 days. This table has to be read only by rows. The table shows average values ± standard errors. Significant differences of enzymatic activities in mycelia fed with the different sugars are indicated by different letters (Tukey HSD test; n = 6). Significant higher activities compared to starvation (no sugar) are indicated in green, lower activities in red bold letters.

S. herbamans	**Glu**	**Fru**	**Suc**	**Glu+Fru**	**Man**	**No sugar**
	Enzymatic activities [nkat/g FW]					
**Aldolase**	0.31 ±0.03	0.23 ±0.02	0.14 ±0.03	0.15 ±0.02	0.32 ±0.02	0 ±0
**Fructokinase**	23.45^{a} ±0.58	15.12^{bc} ±1.95	11.92^{c} ±0.24	13.90^{c} ±0.33	18.64^{b} ±0.55	1.26^{d} ±0.05
**G6PDH**	0.06 ±0.05	0 ±0	0 ±0	0 ±0	0.02 ±0.02	0.01 ±0.01
**GP**	0.2 ±0.03	0 ±0	0.05 ±0.02	0.05 ±0.04	0.17 ±0.05	0.25 ±0.11
**Hexokinase**	24.48^{a} ±0.89	23.40^{a} ±0.98	16.96^{b} ±1.27	17.01^{b} ±1.03	20.62^{ab} ±0.28	18.78^{ab} ±2.13
**Invertase pH 4.5**	178.66^{b} ±1.36	86.69^{c} ±4.42	75.03^{cd} ±1.2	65.47^{cd} ±1.61	21.99^{d} ±1.37	619.66^{a} ±32.14
**PFK**	0.08^{b} ±0.01	0.09^{b} ±0	0.08^{b} ±0.01	0.04^{b} ±0	0.04^{b} ±0.01	0.45^{a} ±0.1
**PGI**	1.04^{b} ±0.11	1.75^{b} ±0.05	1.82^{b} ±0.09	1.31^{b} ±0.05	0.18^{b} ±0.02	168.40^{a} ±1.83
**PGM**	6.82^{d} ±0.11	9.70^{b} ±0.21	11.11^{a} ±0.11	8.07^{c} ±0.08	3.49^{e} ±0.27	6.74^{d} ±0.26
**UGPase**	2.04^{c} ±0.07	9.05^{a} ±0.32	0.95^{d} ±0.09	3.62^{b} ±0.04	9.14^{a} ±0.14	0.27^{d} ±0.04

G6PDH: glucose-6-phosphate dehydrogenase; GP glycogen pyrophosphorylase; PFK: phosphofructokinase; PGI: phosphoglucose isomerase; PGM: phosphoglucose mutase; UGPase: UDP-glucose pyrophosphorylase.

Table S3. Secreted activities of glycolytic enzymes (nkat/gr) in the liquid medium of *S. indica* when cultivated with glucose (Glu), fructose (Fru), sucrose (Suc), an equimolar mix of glucose and fructose (Glu+Fru), mannose (Man) and without sugar for 5 days. The table shows average values ± standard errors. Significant differences of enzymatic activities in mycelia fed with the different sugars are indicated by different letters (Tukey HSD test; *n* = 6). Significant higher activities compared to starvation (no sugar) are indicated in green, lower activities in red bold letters. Activities of UDP-glucose pyrophosphorylase were not detected.

S. indica	**Glu**	**Fru**	**Suc**	**Glu+Fru**	**Man**	**Ara**	**Gal**	**Xyl**	**No sugar**
					Enzymatic activities [nkat/g FW]				
**Aldolase**	0.5 ±0.22	0.29 ±0.11	0.23 ±0.08	0.17 ±0.08	0.91 ±0.69	0.67 ±0.31	0.52 ±0.25	0.54 ±0.22	0.76 ±0.27
**Fructokinase**	3.19^{b} ±0.36	2.04^{b} ±0.59	2.21^{b} ±0.32	2.54^{b} ±0.68	1.49^{b} ±0.29	5.53^{ab} ±1.24	4.93^{ab} ±1.66	5.40^{ab} ±0.83	8.32^{a} ±0.69
**G6PDH**	0.93 ±0.33	0.81 ±0.29	0.85 ±0.26	1.01 ±0.48	1 ±0.2	1.01 ±0.63	1.95 ±0.99	0.99 ±0.61	2 ±0.68
**GP**	7.93^{a} ±1.35	0^{d} ±0	0.36^{c} ±0.21	1.44^{b} ±0.58	0^{d} ±0	0^{d} ±0	0^{d} ±0	8.22^{a} ±2.23	0^{d} ±0
**Hexokinase**	2.39^{ab} ±0.56	2.82^{ab} ±0.23	1.88^{b} ±0.35	2.07^{ab} ±0.41	1.69^{b} ±0.4	5.34^{ab} ±1.14	6.2^{a} ±1.6	5.23^{ab} ±0.99	6.53^{a} ±0.25
**Invertase pH 4.5**	64.41^{bc} ±10.11	63.00^{bc} ±26.93	187.50^{ab} ±68.59	247.82^{a} ±45.07	189.62^{ab} ±48.68	8.88^{c} ±4.17	16.66^{c} ±5.89	16.25^{c} ±5.58	18.68^{c} ±4.35
**PFK**	0.91 ±0.28	0.48 ±0.11	0.44 ±0.08	0.41 ±0.1	1.23 ±0.83	1.23 ±0.34	1.38 ±0.36	1.14 ±0.19	1.76 ±0.63
**PGI**	0.19^{b} ±0.07	0.54^{b} ±0.11	0.53^{b} ±0.14	0.32^{b} ±0.15	1.11^{ab} ±0.46	2.48^{a} ±0.93	1.63^{ab} ±0.46	0.41^{b} ±0.11	1.60^{ab} ±0.32
**PGM**	2.36^{a} ±0.34	0.25^{c} ±0.07	0.26^{c} ±0.08	0.61^{c} ±0.09	0.81^{bc} ±0.63	0.44^{c} ±0.09	0.15^{c} ±0.07	2.26^{ab} ±0.51	0.42^{c} ±0.21

G6PDH: glucose-6-phosphate dehydrogenase; GP glycogen pyrophosphorylase; PFK: phosphofructokinase; PGI: phosphoglucose isomerase; PGM: phosphoglucose mutase.

Table S4. Secreted activities of glycolytic enzymes (nkat/gr) in the liquid medium of *S. herbamans* when cultivated with glucose (Glu), fructose (Fru), sucrose (Suc), an equimolar mix of glucose and fructose (Glu+Fru), mannose (Man) and without sugar for 5 days. The table shows average values ± standard errors. Significant differences of enzymatic activities in mycelia fed with the different sugars are indicated by different letters (Tukey HSD test; *n* = 6). Significant higher activities compared to starvation (no sugar) are indicated in green, lower activities in red bold letters. Activities of UDP-glucose pyrophosphorylase were not detected.

S. herbamans	**Glu**	**Fru**	**Suc**	**Glu+Fru**	**Man**	**Ara**	**Gal**	**Xyl**	**No sugar**
				Enzymatic activities [nkat/g FW]					
**Aldolase**	0.42 ±0.22	0.49 ±0.18	0.41 ±0.1	0.4 ±0.15	0.6 ±0.31	0.27 ±0.02	0.25 ±0.08	0.35 ±0.05	0.11 ±0.05
**Fructokinase**	2.89 ±0.83	2.42 ±0.72	2.11 ±0.17	2.44 ±0.58	2.68 ±0.38	2.46 ±0.29	1.92 ±0.42	2.13 ±0.35	1.3 ±0.14
**G6PDH**	0.24 ±0.1	0.35 ±0.14	0.13 ±0.06	0.12 ±0.04	0.31 ±0.2	0.11 ±0.08	0.06 ±0.05	0.08 ±0	0.2 ±0.14
**GP**	**7.19^{a}** ±2.27	0.40^{b} ±0.2	1.98^{b} ±0.32	2.38^{ab} ±1.02	1.27^{b} ±0.72	0.14^{b} ±0.11	0.25^{b} ±0.21	2.85^{ab} ±0.92	0.10^{b} ±0.07
**Hexokinase**	2.44 ±0.77	2.84 ±0.68	1.71 ±0.22	1.77 ±0.24	3.42 ±0.41	2.16 ±0.45	1.68 ±0.27	2.56 ±0.48	1.35 ±0.18
**Invertase pH 4.5**	308.54^{b} ±122.46	911.83^{ab} ±132.03	826.20^{ab} ±150.53	850.24^{ab} ±160.69	**1034.29^{a}** ±188.68	338.05^{b} ±76.8	392.78^{b} ±80.93	643.84^{ab} ±116.63	410.12^{b} ±53.52
**PFK**	0.99^{ab} ±0.16	0.86^{ab} ±0.15	0.84^{ab} ±0.12	0.79^{ab} ±0.16	**1.35^{a}** ±0.29	0.64^{ab} ±0.16	0.49^{b} ±0.13	0.72^{ab} ±0.08	0.37^{b} ±0.05
**PGI**	0.85 ±0.63	2.53 ±1.01	1.13 ±0.27	1.06 ±0.24	2.21 ±0.78	1.16 ±0.23	1.06 ±0.21	0.86 ±0.19	1.37 ±0.73
**PGM**	**1.55^{a}** ±0.26	0.50^{b} ±0.24	0.65^{b} ±0.11	0.54^{b} ±0.16	0.47^{b} ±0.14	0.24^{b} ±0.05	0.14^{b} ±0.06	0.81^{ab} ±0.24	0.08^{b} ±0.03

G6PDH: glucose-6-phosphate dehydrogenase; GP glycogen pyrophosphorylase; PFK: phosphofructokinase; PGI: phosphoglucose isomerase; PGM: phosphoglucose mutase.
